# The effectiveness of transcutaneous cervical ultrasonography for diagnosing peritonsillar abscess in a patient complaining of sore throat

**DOI:** 10.1002/jgf2.364

**Published:** 2020-08-27

**Authors:** Hiroshi Hori, Takahiko Fukuchi, Hitoshi Sugawara

**Affiliations:** ^1^ Department of Comprehensive Medicine 1 Division of General Medicine Saitama Medical Center Jichi Medical University Saitama Japan

**Keywords:** peritonsillar abscess, point‐of‐care transcutaneous cervical ultrasound, sore throat

## Abstract

Peritonsillar abscess should be considered as a differential in patients presenting with fever, sore throat, and other cold‐like symptoms. Point‐of‐care transcutaneous cervical ultrasound is effective for diagnosing peritonsillar abscess in place of computed tomography (CT) imaging. Tongue movement during ultrasound examination will help confirm the presence of anatomical lesions on tonsils, and comparing the affected and unaffected sides will improve proper diagnosis.

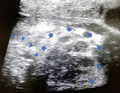

A febrile 19‐year‐old man experiencing pharyngeal pain presented to our emergency outpatient department; his body temperature was 40°C. A transcutaneous cervical ultrasonography revealed swelling of the left palatine tonsil with a hypoechoic mass measuring approximately 25 mm (Figure 1A). No abnormalities were identified in his right palatine tonsil (Figure 1B). Computed tomography (CT) revealed a low‐density region in the left palatine tonsil measuring 26 mm × 22 mm, surrounded by a contrast‐enhanced area (figure 2); thus, we established a diagnosis of peritonsillar abscess (PTA). After drainage and administration of sulbactam sodium/ampicillin sodium at 12 g/day, his body temperature was normalized, and the pain ameliorated rapidly. A culture from the abscess identified *Fusobacterium necrophorum*.

**FIGURE 1:**
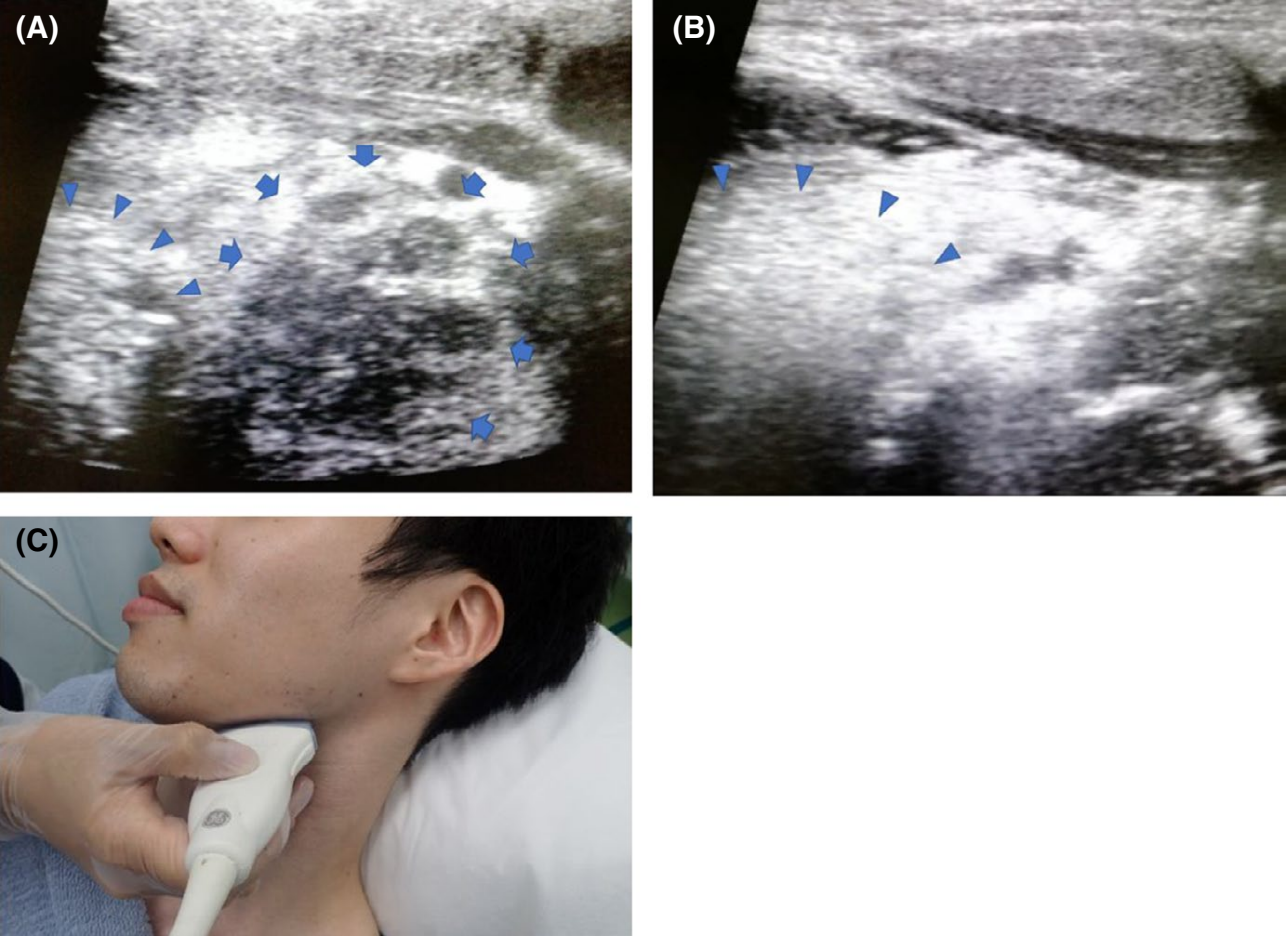
The patient was asked to move their tongue (arrowhead) to improve imaging of the tonsil. A swollen palatine tonsil and low‐intensity abscess measuring 25 mm in size (arrow) can be seen behind the tongue (A, Video [Link], [Link]). Ultrasonography image of the unaffected side; the right palatine tonsil is not swollen (B, Video [Link], [Link])

**FIGURE 2:**
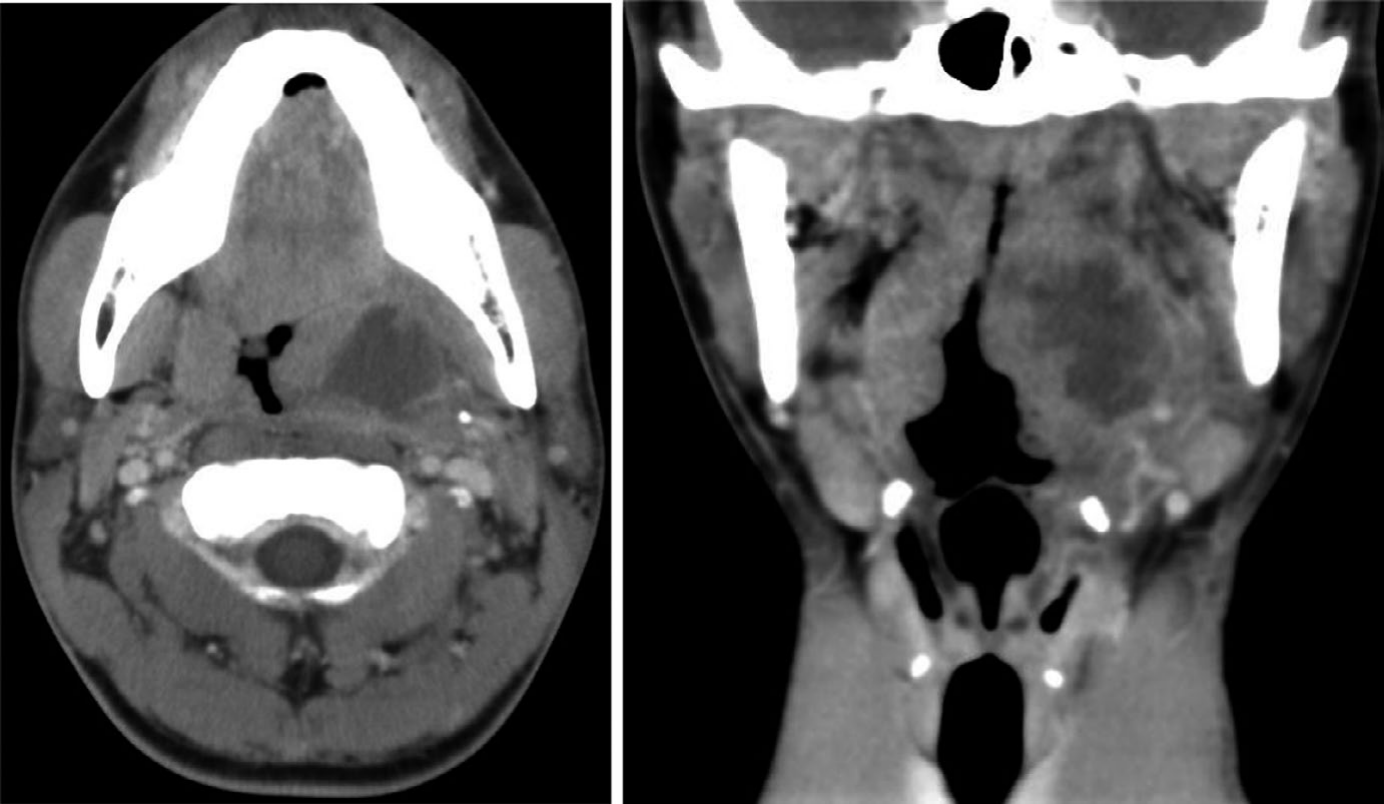
Contrast‐enhanced computed tomography (CT) of the lateral side of the left tonsil revealed a low‐density margin of contrast enhancement measuring 26 mm × 22 mm

PTA is the most common type of deep neck abscess.^1^ The primary symptoms of PTA are similar to a common cold and include fever, unilateral severe sore throat, and painful swallowing. Delayed diagnosis can result in serious complications, such as airway obstruction, sepsis, and Lemierre's syndrome.^1^ It is difficult to distinguish peritonsillar cellulitis from PTA using only *physical examination*.^2^ Contrast‐enhanced CT scans are effective; however, they incur high costs, involve radiation exposure, and are difficult to obtain in children. Alternatively, ultrasonography is minimally invasive, cost effective, can be performed rapidly, and involves no radiation exposure. Ultrasound methods include intraoral ultrasonography (IOU) and transcutaneous cervical ultrasonography. Transcutaneous cervical ultrasonography has a sensitivity of 80%‐91%, a specificity of 80%‐93%,^3^ and is useful for PTA diagnosis; visualization of the jugular also enables evaluation of thrombotic complications (Lemierre's syndrome).

Techniques for imaging a peritonsillar abscess:

1. Use a high‐frequency linear transducer (6‐12 MHz).
2. Aim the probe at the lower jaw, parallel to the line connecting the ear and jaw (Figure 1C).
3. Delineate the lower jawbone and submandibular gland, and then change the angle of the probe to delineate the tongue and tonsils behind. Having the patient move their tongue enables the palatine tonsils to be easily identified.
4. PTA is suspected in palatine tonsils that have swollen to more than 20 mm in size and present with a low‐intensity structure;^3^ doppler waves do not enter the abscess. Since the size of the palatine tonsils differs between individuals, it is important to compare it with the unaffected side.

Transcutaneous cervical ultrasonography is useful in PTA diagnosis. Skill is required to identify and visualize the lesion.

## CONFLICT OF INTEREST

The authors have stated explicitly that there are no conflicts of interest in connection with this article.

## INFORMED CONSENT

We have obtained written consent from the patient for publication of the report.

## Supporting information

Video S1

Video S2

App S1
