# Supplementary material for: The effectiveness of transcutaneous cervical ultrasonography for diagnosing peritonsillar abscess in a patient complaining of sore throat
Source: J Gen Fam Med. 2020 Aug 27;22(1):47–8. doi: 10.1002/jgf2.364 (PMC7796778; doi:10.1002/jgf2.364)
Supplement: Supplementary file 3 — App S1 [file JGF2-22-47-s003.docx]

Supporting information

Figure videos A, B

The patient was asked to move their tongue. Behind the tongue, a swollen palatine tonsil and low-intensity abscess measuring 25 mm in size (arrow) can be seen (Supp video A). Ultrasonography image of the unaffected side (Supp video B)
